# Mercury pollution in vegetables, grains and soils from areas surrounding coal-fired power plants

**DOI:** 10.1038/srep46545

**Published:** 2017-05-09

**Authors:** Rui Li, Han Wu, Jing Ding, Weimin Fu, Lijun Gan, Yi Li

**Affiliations:** 1 College of Horticulture, Nanjing Agricultural University, Nanjing, 210095, P. R. China; 2 College of Life Sciences, Nanjing Agricultural University, Nanjing, 210095, P. R. China; 3 Department of Plant Science and Landscape Architecture, University of Connecticut, Storrs, CT 06269, USA

## Abstract

Mercury contamination in food can pose serious health risks to consumers and coal-fired power plants have been identified as the major source of mercury emissions. To assess the current state of mercury pollution in food crops grown near coal-fired power plants, we measured the total mercury concentration in vegetables and grain crops collected from farms located near two coal-fired power plants. We found that 79% of vegetable samples and 67% of grain samples exceeded the PTWI’s food safety standards. The mercury concentrations of soil samples were negatively correlated with distances from the studied coal-fired power plants, and the mercury contents in lettuce, amaranth, water spinach, cowpea and rice samples were correlated with the mercury contents in soil samples, respectively. Also, the mercury concentrations in vegetable leaves were much higher than those in roots and the mercury content of vegetable leaves decreased significantly after water rinses. Our calculation suggests that probable weekly intake of mercury for local residents, assuming all of their vegetables and grains are from their own farmland, may exceed the toxicologically tolerable values allowed, and therefore long-term consumptions of these contaminated vegetables and grains may pose serious health risks.

Rapid industrial development in China is highly dependent on coal energy. Coal comprises ~70% of the energy supply in China, which is much higher than the global average of 28%^1^. In 2012, China consumed 1,785.3 million tons of coal, constituting more than half (50.2%) of the total global coal consumption^2^. This has had severe environmental consequences in China, including thick smog in Beijing and many other cities, exacerbating the greenhouse effect, and widespread heavy metal pollution in the air, water, soil, and agricultural products.

Mercury is a particularly important heavy metal to consider when examining the environmental consequences of coal burning. Although mercury is released into the environment from natural and anthropogenic sources, coal-fired power plants have been identified as the largest source of mercury emissions^2^. In 1995, the total mercury emissions from coal-fired boilers in China based on mercury emission factors was 302.87 tons, and ~2,493.8 tons of mercury was released into the environment from coal combustion between 1978 and 1995^3^.

Mercury emitted from coal-fired boilers in power plants has increased mercury pollution in neighboring areas. It has been reported that in the atmosphere, mercury is in gaseous and particulate forms^4^,^5^,^6^. Plants can absorb mercury that is deposited on leaf surfaces^7^,^8^,^9^. Besides, plants can also uptake mercury from water and soil via roots^10^. Majority of mercury accumulates locally in the plant with little mobility, and only small portions may be released into the atmosphere or transported to other plant organs^7^,^8^,^11^,^12^. Mercury accumulated in plants are in the forms of Hg(0), Hg(II), and organic Hg, and usually aquatic plants contain more methyl mercury (organic Hg) than terrestrial plant^13^,^14^. On the other hand, the mercury that accumulates in fish is predominantly organic methyl Hg^15^,^16^. However, further investigations are needed to further determine how plants uptake and absorb mercury, and how important the dry deposition of fly ash or the uptake of gaseous Hg are for higher plants.

Mercury can be harmful at very low concentrations because of its high toxicity and ability to bioaccumulate^17^,^18^,^19^. The mercuric ion is one of the strongest thiol-binding agents, and mercury absorbed into the human body attaches to thiol residues in proteins, making it difficult to eliminate from living organisms^19^. Intracellular mercury can inactivate sulfur, which can inhibit various enzymes, cofactors, and hormones and result in many diseases in animals or human^19^. Mercury can build up and accumulate in the human body and cause severe neurological disorders in children and adults, and also harm unborn fetus if the mother already has a high MeHg level in the body^17^. One of the most debilitating diseases caused by mercury exposure is Minamata disease. In 1955, inhabitants of Minamata Bay, Japan, who consumed mercury-contaminated fish and seafood suffered from mercury poisoning, which particularly damaged patients’ nervous systems. As a result, at least 439 people died of Minamata disease^15^,^16^. The Provisional Tolerable Weekly Intake (PTWI) of mercury suggested by The World Health Organization (WHO) is 1 μg/kg body weight^20^, and the maximum mercury concentration defined by the Food Safety Standards used in China is 10 μg/kg in vegetables and 20 μg/kg in grains (Maximum Levels of Contaminants in Foods, GB 2762-2012)^21^.

Other researchers have investigated mercury concentrations in vegetables and grains cultivated near various sources of mercury pollutions, including mercury mines^22^,^23^, zinc plants^24^, fluorescent lamp factories^25^, geothermal power plants^26^, chlor-alkali plants^27^, industrial zones^28^, coal mines^29^, and oil wells^30^. It has also been reported that vegetables and grains from coal or mercury mining regions are heavily contaminated with mercury. For instance mercury concentrations of biological samples collected from the Kaili coal mining region in China was 883 μg/kg^29^ and from the Idrija mercury mine area in Slovenia was 12,713 μg/kg^23^. Although coal-fired power plants represent the largest source of mercury emissions in many regions of the World^2^, the current state of mercury levels in vegetable and grain crops grown near such power plants has not been assessed.

In this study, we investigated the mercury contamination in grains and popular vegetables cultivated near two coal-fired power plants, estimated the mercury intake of local residents, and discussed potential health risks associated with mercury consumption. This is the first study to investigate mercury concentrations in vegetables and grains cultivated near coal-fired power plants.

## Results and Discussion

### Soil near coal-fired power plants is heavily polluted with mercury

**Figure 1:**
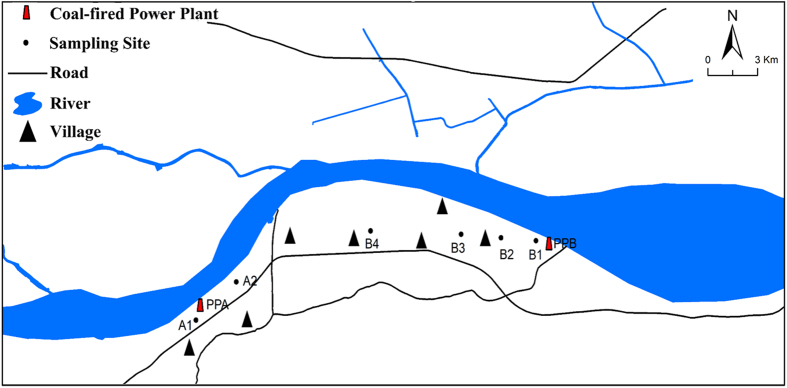
Plant and soil sampling sites around Power Plant A and Power Plant B. Black triangle: villages. PPA: Power Plant A; PPB: Power Plant B; A1: 1 km from Power Plant A; A2: 3 km from Power Plant A; B1: 1 km from Power Plant B; B2: 3 km from Power Plant B; B3: 5 km from Power Plant B; B4: 10 km from both Power Plants A and B.

**Figure 2:**
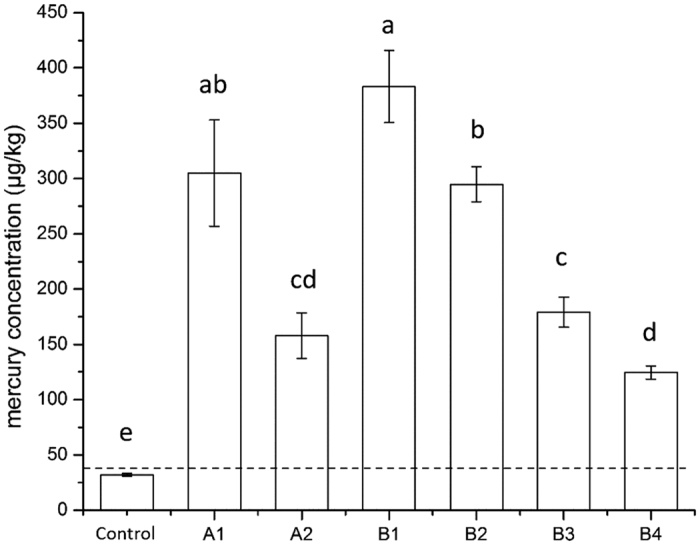
Mercury concentrations in soil samples collected near the power plants. Dashed line: background soil mercury concentration in China (37 μg/kg)^32^. Sampling sites are marked in Fig. 1. Uncontaminated soil samples were collected from a farmland site >55 km away from any power plants. A1, A2, B1, B2, B3 and B4 are locations where biological samples were collected. The level of significance was defined at P < 0.05 using T-test.

We measured mercury concentrations in soil samples collected from six field locations (Locations A1, A2, B1, B2, B3, and B4 in Fig. 1) located within 10 km of two coal-fired power plants (A and B). The average soil mercury concentrations were 305.10 ± 47.97 μg/kg at A1 (1 km from Power Plant A), 157.81 ± 20.52 μg/kg at A2 (3 km from Power Plant A), 383.23 ± 32.59 μg/kg at B1 (1 km from Power Plant B), 294.91 ± 15.67 μg/kg at B2 (3 km from Power Plant B), 179.14 ± 13.53 μg/kg at B3 (5 km from Power Plant B), and 124.58 ± 6.14 μg/kg at B4 (10 km from both Power Plants A and B) (Fig. 2). The mercury concentrations in the soil samples were negatively correlated with their distances from the sites of the two studied coal-fired power plants (R2 = 0.82, P < 0.001, Supplemental Figure 1). Our results are consistent with the finding of Filippelli *et al*.^3^ who examined spatial distribution of soil Hg as a function of distance from a coal-fired power plant in the US.

The average mercury concentrations of the soil samples from the two power plant regions were more than 10 times higher than these of soils from a control site (32.01 ± 1.30 μg/kg, 55 km away from Power Plant A) and the background soil mercury concentrations in China is about 37 μg/kg^32^ (Table 1). These results indicate that the soils around coal-fired power plants was heavily polluted. Also as shown in Table 1, it is reasonable to predicate that the mercury concentrations of surrounding soils will increase, if these two power plants continue to operate. The average mercury concentrations of soil samples collected 1 km away from Power Plant Baoji (606 μg/kg)^33^, Power Plant “A Horizon” (19,900 μg/kg)^34^, and Power Plant Chengdu (24,546 μg/kg)^35^ were much higher than those of Power Plants A (305 μg/kg) and B (383 μg/kg) in this research (Table 1). The main reason for this discrepancy should be that these three power plants have been operated for much longer than the two for this study. Power Plant A has been in operation for 4 years and Power Plant B has been in operation for 3 years, while Power Plant Baoji has been in operation for 16 years^33^, Power Plant “A Horizon” has been in operation for ~30 years^34^, and Power Plant Chengdu has been in operation for >30 years^35^.

Soil mercury contents may be reduced naturally via vaporization and run-off. However, rates of these processes are not well studied. There are many factors^36^,^37^,^38^ such as forms of mercury (e.g., ionic, organic or inorganic), soil pH, temperature, vegetative cover, and rain fall can all influence these processes. Thus, soil’s mercury retention rates can be different from one location to another. When determining soil’s mercury retention rates, all of these factors should be taken into consideration.

### Vegetable and grain crops grown near coal-fired power plants are heavily polluted with mercury

We investigated the mercury content of the edible parts of ten types of vegetable and grain crops from the selected locations in Fig. 1. The samples collected from a grocery store which is far from any power plants (>55 km) were used as the un-contaminated control to compared with samples from the coal-fired power plant areas. The average mercury concentrations in lettuce leaves were 21.03 ± 0.16 μg/kg at B1, 19.41 ± 1.16 μg/kg at B2, 9.17 ± 0.52 μg/kg at B3, and 7.23 ± 0.57 μg/kg at B4. Based on these results, the mercury concentrations in lettuce leaves were positively correlated with those of corresponding soil samples and negatively correlated with the distances from the site of the power plants (Supplemental Fig. 1). This is also the case for amaranth leaves, water spinach leaves, cowpea, and rice grains (P < 0.01), and also tomato, eggplant and cucumber fruits (Supplemental Table 1).

The mercury contents in 79% of vegetable samples and 67% of grain samples exceeded the maximum allowed mercury levels defined by the Food Safety Standards in China [10 μg/kg fresh weight (FW) for vegetables and 20 μg/kg FW for grains according to Maximum Levels of Contaminants in Foods, GB 2762-2012]^21^, and the highest mercury concentrations measured in the vegetable and grain samples were 8.6 and 6.3 times higher than the allowed levels, respectively. Meanwhile, none of the vegetable and grain samples purchased from a grocery store >55Km away from any coal-fired power plant exceeded the maximum levels allowed (Table 2).

Comparing the mercury concentrations in different types of vegetables and grains, we found that the mercury contents in the edible parts of lettuce, amaranth, water spinach, tomato, eggplant, pepper, cucumber, and cowpea were 2.1, 2.9, 5.4, 7.6, 4.3, 6.2, 1.8, and 5.7 fold greater than the maximum allowed mercury levels, respectively^21^. In addition, the mercury concentrations in the rice and maize seed samples were 3.0 and 2.1 fold higher than the maximum allowed mercury level in grains, respectively (Maximum Levels of Contaminants in Foods, GB 2762-2012)^21^ (Table 2). In the three leafy vegetables (i.e., lettuce, amaranth, and water spinach), water spinach contained the highest mercury concentration. Of the tested fruits (i.e., tomato, eggplant, pepper, cucumber, and cowpea), tomato had the highest mercury concentration. Of the grain samples, rice contained significantly more mercury than maize (Table 2). Based on these results, the mercury content differed significantly among different vegetable and grain crops, and the differences in mercury concentration among different plants may be due to species-specific metal absorption and accumulation properties^39^. This type of information can help farmers to choose crop plant species that accumulate relatively low amounts of mercury.

**Figure 3:**
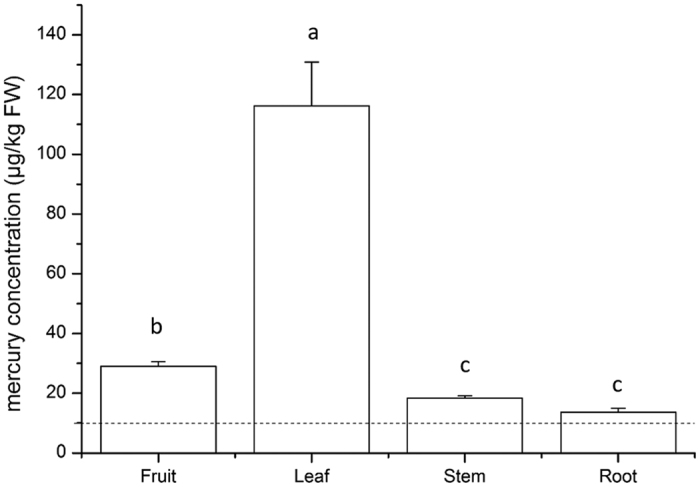
Mercury distribution in organs of tomato grown in Location B3. Dashed line: maximum allowed mercury level in vegetables (10 μg/kg FW) (Food Safety Standard in China, GB 2762-2012)^21^. Tomato tissue samples were collected from Location B3 located 5 km from Power Plant B. The level of significance was defined at P < 0.05 using T-test.

To investigate mercury accumulation in different plant organs, we measured the mercury concentrations in leaves, fruit, stems, and roots of tomatoes grown in Location B3. The mercury concentrations were 116.17 ± 14.69 μg/kg in leaves, 29.07 ± 1.45 μg/kg in fruits, 18.35 ± 0.83 μg/kg in stems, and 13.64 ± 1.37 μg/kg in roots. Mercury concentrations were much higher in leaves than in fruits, and the mercury concentrations in the aboveground organs were higher than those in roots (Fig. 3).

Previous studies have demonstrated that plants can absorb mercury from both air and soil. When plants absorb mercury mainly from the soil, the mercury content should be higher in roots, while the mercury contents should be higher in shoots and leaves tissues if air mercury is the main source of mercury in plants^40^,^41^. In our studies, the mercury contents were much greater in leaves than in roots (Fig. 3), indicating that the source of the mercury in the plant samples collected near Power Plants A and B should be mainly from the air. This is because the two coal-fired power plants have been operated for less than 5 years and therefore the air mercury is the main source of the mercury in the plants. However, our results cannot distinguish how much was from air or soil. Future study may be done using a plastic barrier to determine how much mercury in plants is absorbed via roots and how much is from leaf surface depositions.

Results published previously by others show that mercury concentrations in vegetables and grains from coal-fired power plant regions are higher than those of samples from zinc plant, oil well, and fluorescent lamp factory regions (Table 3). In China, there are thousands of coal-fired power plants and most of them are located in densely populated eastern regions, particularly in suburbs where vegetables for residents in cities are produced^2^,^42^. Thus, mercury generated from coal-fired power plants may cause potential health risks for the people living surrounding coal-fired power plants.

### Effects of vegetable washing and processing on mercury contents

It has been reported that the mercury in fly ash is predominantly in the form of particulate mercury that can be deposited on plant leaf surfaces due to wet and dry deposition plants^4^,^5^. The amount of mercury that can be washed off from vegetables we have analyzed should be those adhering to leaf and shoot surfaces. Therefore, we compared the mercury concentrations of vegetable leaves before and after washing to investigate how much mercury on the leaf surfaces can be eliminated with water rinses. We selected lettuce and amaranth for the determination because they are popular leafy vegetables in many areas of China and the World. Table 4 shows the mercury contents of water-rinsed vegetable samples were reduced, with 19-63% reductions observed in lettuce and amaranth leaf samples. The difference in the mercury content in amaranth leaves before and after water rinses was 26.77 μg/kg in Location B1, 13.00 μg/kg in Location B2, 9.39 μg/kg in Location B3, and 0.83 μg/kg in Location B4 (Table 4), demonstrating that water rinse can significantly reduce mercury contents in the tested leafy vegetables.

The majority of China’s coal-fired power plants uses limestone-gypsum wet flue gas desulfurization systems to remove sulfur from the flue gas^43^, including the two power plants in this study. However, after the desulfurization process, flue gas contains substantial amounts of gypsum and other suspended fly ash^44^. Fly ash is an important vehicle for particulate mercury because the mercury can be absorbed by fly ash in flue gas cannot easily diffuse into the atmosphere. The mercury containing ash is usually deposited onto land and plant leaf surfaces through dry and wet deposition^6^. Reduction in release of fly ash from coal-fired power plants may represent one strategy to reduce mercury contamination of plants and soil.

Because elemental mercury can easily evaporate at elevated temperatures, cooking may therefore reduce mercury in food. However, studies have shown that cooking reduced mercury contents in mushroom by 10% and had no effect on the mercury contents in fish^45^,^46^,^47^. These studies suggest that cooking-mediated reductions in mercury content in food may be minimal, consistent with the reports that inorganic mercury and methyl mercury irreversibly bind to cellular components such as thiol-containing proteins in cells^17^,^48^.

### Potential health risk to inhabitants via consumption of mercury-polluted vegetables and grains

We estimated probable weekly intake (PWI) of mercury by residents who rely on locally produced produces using Equations (1) and (2) described by Miklavčič^22^. The 95^th^ percentile of mercury concentrations found in vegetable and grain samples were used in the calculation^22^. In China, most farmers produce and consume their own vegetables, fruits, grains, and meats because of higher costs for grocery store products and also poor transportation accessibility^49^. We therefore assume that the residents around coal-fired power plants obtain more than 95% of their food (vegetables, grains and meats) derived from their polluted farmland, and this assumption has been also used in other studies^22^,^24^,^25^,^27^. Our calculations were based on the 95^th^ percentile of Hg concentration, that were 71 ng/g FW for vegetables and 62 ng/g FW for rice samples. Because rice is the main food grain in China, while maize is mainly used as livestock feed^50^, we used our mercury data from rice for the calculations. Also, it has been reported that an average consumption for vegetables is 301 g/person/day and for grains is 217 g/person/day in China^24^,^50^,^51^. For 2010, for instance, the data from the Food and Agriculture Organization (http://faostat.fao.org) show that the total vegetable consumption in China was 332.20 kg/person. In addition, the average bodyweight of Chinese adults is 55.9 kg^51^,^52^. Using these data, the estimated PWIs of mercury for the local residents having all vegetables and grains from their own farmland are 2.674 and 1.687 μg/kg body weight (bw)/week, with 4.36 μg/kg bw for total weekly mercury intake, which is several fold higher than the PTWI’s upper limit, 1 μg/kg bw, according to Provisional Tolerable Weekly Intake guidelines (Table 5). This suggests potential health risks for local residents largely relying on locally produced vegetables and rice. Further, mercury contamination in vegetables and grains grown in the areas in this study will likely increase in the future due to increasing mercury accumulation in the soil over time as shown previously^53^.

## Conclusions

Numerous studies have shown that vegetables and grains cultivated near various mercury sources can be contaminated. Coal-fired power plants are the largest sources of mercury released to the environment in China, and we reported that soil, vegetable, and grain samples collected from field locations within 10 km distance from Power Plant A and B had significantly higher mercury concentrations than the samples purchased from a grocery store away from any power plant, with 79% of vegetable samples and 67% of grain samples exceeded the upper limit of allowable mercury level (Maximum Levels of Contaminants in Foods, GB 2762-2012)^21^. We also showed that mercury contents of vegetable leaves decreased significantly if fly ash was rinsed off from leaf surfaces. Further, our calculations suggest that there may be mercury-mediated health risks for the local residents if all of their vegetables and grains are from their own farmland.

## Methods

### Study areas

The average Hg concentration in coal used in power plants from this area is 120 μg/kg^54^. Power Plant A began operation in 2012. Based on the data from the power plant company’s website, the main generating units in Power Plant A are two 1030 MW coal-fired generating units that use 285 g of coal per 1 kWh of electricity, and in 2013, Power Plant A produced 12.8 billion kWh of electricity. Power Plant A consumed ~3.648 Mt coal and emitted ~437.8 kg of mercury in the year 2013.

Power Plant B began operation in 2013. From the data from the company’s website, the main generating units are two 660 MW coal-fired generating units that use 290.78 g coal per kWh electricity, and in 2013, Power Plant B produced 7.7 billion kWh of electricity. Approximately 268.7 kg of mercury was emitted from the coal burned (~2.239 Mt) in the year 2013.

### Sampling and pretreatment

Vegetables, grains, and soil (0–15 cm deep) samples were collected in six open field locations (A1, A2, B1, B2, B3, and B4) close to Power Plants A and B (Fig. 1) in 2015. We sampled lettuce, amaranth, and water spinach as typical leafy vegetables; tomato, eggplant, pepper, cucumber, and cowpea as typical fruit vegetables; and rice and maize as typical grains.

The fresh vegetable and grain samples were stored in plastic bags for transport. Samples were treated in the laboratory as described previously^24^. The fresh vegetable and grain samples were flushed with tap water for 10 min and then washed three times with Milli-Q water. Each individual vegetable sample was separated into root, stem, leaf, and seed or fruit sub-samples. The fresh weights of these sub-samples were recorded, which were then dried at 55 °C. The dried sub-samples were weighed to record their dry weight. Then, the sub-samples were ground into fine powder and stored in polythene tubes for further analysis. Samples used to investigate the influence of fly ash on mercury concentrations were pretreated following the same methods as described above, except they were not washed with water. Control vegetable and grain samples were collected from a grocery store located >55 km from the two power plants. We pooled vegetable and grain samples of the same type from the same field into a pooled sample containing ~20 individual plants. For each type of vegetable or grain in each field, parallel pooled samples were measured. Soil samples were air-dried, crushed, and passed through a 0.18-mm mesh sieve and the treated soil samples were stored in polythene tubes for further analysis. Control soil samples were collected from a farm located >55 km from the two power plants. Soil samples from the same field were pooled into a pooled sample containing ~20 individual samples. For each soil sample in each field, parallel pooled samples were measured.

### Determination of mercury in vegetable, grain, and soil samples

Dried, pulverized vegetable or grain samples were placed into Teflon vessels, and 10 mL of HNO_3_ was added to each sample. Dried, pulverized soil samples were placed into Teflon vessels, and 9 mL of HCl and 3 mL of HNO_3_ were added to each sample. The sample digestions were based on EPA Method 7473^55^ and accomplished in a Microwave Sample Preparation System (ETHOS One; Milestone, Sorisole, Italy).

After digestion, the vessels were opened carefully. The final solutions were cooled and transferred into 50-mL calibrated flasks, and their volumes were completed with deionized water. All sample processing was performed in a laminar flow fume cupboard to avoid external contamination. The mercury concentrations of all soil and vegetable samples were analyzed using atomic fluorescence spectrophotometry (AFS-230E; HG, Shaanxi, China) after pre-concentration and dilution^56^,^57^. Reagent blanks and internal standards were used when appropriate to ensure accuracy and precision. The results were analyzed with analyses of variance.

### Exposure assessment

To estimate local residents’ potential exposure to mercury via vegetable and grain consumption, Equations (1) and (2) were used to calculate the PWI of mercury^22^:


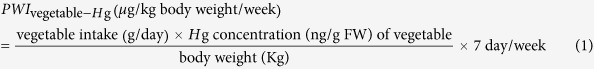



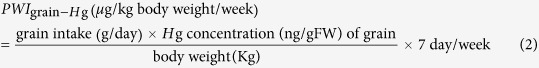


Hg concentration of vegetable and grain samples used in the two equations were the 95^th^ percentile of mercury concentrations in a statistical analysis of all vegetable and grain sample data, respectively^22^. Therefore Hg concentration was 70.83 ng/g FW for vegetables and 62.02 ng/g FW for grains. The average annual vegetable and grain consumption for adults in China is 301.1 g/person/day and 217.6 g/person/day, respectively^24^,^50^,^51^. The average adult bodyweight is 55.9 kg in China^51^,^52^.

## Additional Information

**How to cite this article:** Li, R. *et al*. Mercury pollutions in vegetables, grains and soils from areas surrounding coal-fired power plants. *Sci. Rep.* **7**, 46545; doi: 10.1038/srep46545 (2017).

**Publisher's note:** Springer Nature remains neutral with regard to jurisdictional claims in published maps and institutional affiliations.

## Supplementary Material

Supplementary Information

## Figures and Tables

**Table 1:** Mercury concentrations of soil samples from the two power plants.

Sampling site	Average mercury level* (μg/kg)	Maximum Mercury level (μg/kg)	Years of operation	Reference
Power Plant A, this study	305 ± 47.97	362	4	
Power Plant B, this study	383 ± 32.59	407	3	
Power Plant BaoJi, China	606	2,105	16	33
Power Plant “A Horizon”, US	19,900	—	30	34
Power Plant ChengDu, China	24,546	40,032	>30	35

Dashed line: background soil mercury concentration in China (37 μg/kg)32. Sampling sites are marked in Fig. 1. Uncontaminated soil samples were collected from a farmland site > 55 km away from any power plants. A1, A2, B1, B2, B3 and B4 are locations where biological samples were collected. The level of significance was defined at P < 0.05 using T-test.

**Table 2:** Mercury concentrations in vegetables and grains grown near coal-fired power plants.

		Mercury concentration (μg/kg Fresh Weight)						
Samples		Location A1	Location A2	Location B1	Location B2	Location B3	Location B4	Control Sample^#^
Leafvegetables	Lettuce	39.04 ± 4.41*	22.70 ± 1.81*	21.03 ± 0.16*	19.41 ± 1.16*	9.17 ± 0.52*	7.23 ± 0.57*	0.35 ± 0.10
	Amaranth	46.40 ± 2.33*	27.76 ± 1.13*	29.29 ± 5.06*	7.50 ± 0.21*	5.52 ± 0.86*	3.64 ± 0.37*	0.28 ± 0.21
	Water spinach	86.69 ± 2.16*	69.02 ± 5.17*	54.46 ± 4.55*	49.19 ± 0.28*	38.97 ± 3.43*	23.88 ± 1.28*	0.85 ± 0.22
FruitVegetables	Tomato	71.80 ± 11.95*	29.80 ± 3.03*	76.33 ± 5.47*	57.09 ± 8.33*	**29.07 ± 1.45**	9.79 ± 0.43*	0.73 ± 0.36
	Eggplant	42.37 ± 4.24*	13.07 ± 1.73*	43.36 ± 1.71*	25.02 ± 1.80*	14.61 ± 2.95*	3.25 ± 0.41*	0.43 ± 0.39
	Pepper	49.66 ± 1.40*	14.65 ± 1.63*	62.09 ± 3.22*	30.89 ± 2.19*	15.75 ± 1.27*	4.69 ± 0.13	0.93 ± 0.84
	Cucumber	38.45 ± 1.40*	9.87 ± 0.11*	18.21 ± 1.19*	16.94 ± 0.66*	10.09 ± 0.40*	2.18 ± 0.34*	0.87 ± 0.24
	Cowpea	56.31 ± 4.03*	11.63 ± 1.36*	57.30 ± 9.24*	21.75 ± 1.55*	18.46 ± 0.61*	11.20 ± 0.95*	0.93 ± 0.13
Grain	Rice	62.95 ± 3.88*	29.24 ± 2.04*	59.21 ± 4.36*	43.30 ± 2.19*	37.15 ± 2.39*	24.99 ± 1.99*	0.55 ± 0.48
	Maize	21.02 ± 1.98*	6.68 ± 1.002*	21.18 ± 0.67*	4.68 ± 0.707*	1.06 ± 0.08*	0.55 ± 00.63	0.72 ± 0.19

Sampling sites are marked in Fig. 1. ^#^Control samples were collected from a grocery store > 55 km from Power Plant A. Bolded numbers indicate that the mercury concentration in samples exceeded the maximum allowed mercury level of 10 μg/kg FW in vegetables and 20 μg/kg FW in grains (food safety standard in China, GB 2762–2012)^21^. *The mercury concentration between the same kind of vegetable and grain samples collected from coal-fired power plant regions and grocery store was significantly different at P = 0.05 level.

**Table 3:** Mercury contents in vegetables grown near sources of mercury emissions.

Sampling site	Range (μg/kg)	study area (km)	Reference
Power Plant A, this study	9.87–86.69 FW	3	
Power Plant B, this study	2.18–76.33 FW	10	
Zinc Plant-Huludao, Shandong, China	0.5–15 FW	6	24
Oil well, Niger Delta, Nigeria	2–17 FW	7	30
Industrial zone, Tehran, Iran	18–24 FW	3	28
Fluorescent lamp factories, GaoHong, China	3.2–47.8 FW	10	25
Chlor-alkali plant, Rosignano Solvay, Italy	0.05–111 DW	40	27
Geothermal Power Plants, Piancastagnaio, Italy	5–210 FW	5	26
Coal Mining- Kaili, Guizhou, China	335–883 FW	15	29
Mercury Mine, Idrija, Slovenia	53–12713 DW	2	22

**Table 4:** Effects of water-rinses on mercury content in vegetable leaves.

Samples	Mercury concentration (μg/kg Fresh Weight)							
	Location B1		Location B2		Location B3		Location B4	
	Unwashed	Washed	Unwashed	Washed	Unwashed	Washed	Unwashed	Washed
Lettuce	**35.70 ± 1.47**	21.03 ± 0.16^*^	**33.94 ± 4.6**	19.41 ± 1.16^*^	**23.02 ± 5.27**	9.17 ± 0.52^*^	13.15 ± 1.68	7.24 ± 0.57^*^
Amaranth	**56.06 ± 2.96**	29.29 ± 5.06^*^	**20.5 ± 0.47**	7.50 ± 0.21^*^	**14.91 ± 0.17**	5.52 ± 0.86^*^	4.47 ± 0.38	3.64 ± 0.37

Bolded numbers indicate that the mercury concentration in samples exceeded the maximum allowed mercury level of 10 μg/kg FW in vegetables (GB 2762–2012)21. The sampling sites are marked in Fig. 1. *The mercury concentration between the same vegetable and grain samples before and after washed was significantly different at P = 0.05 level.

**Table 5:** Probable weekly intake of mercury via vegetable and grain consumption in residents living near coal-fired power plants.

	Mercury concentration (μg/kg FW)	PWI (μg/kg bw/week)
Mercury intake from vegetables	70.83	2.67
Mercury intake from grains	62.02	1.69
Total intake from vegetables & grains		4.36

PWI: probable weekly intake of mercury; PTWI: Provisional Tolerable Weekly Intake of mercury recommended by the World Health Organization (1 μg/kg bw)^20^. The local residents, assuming their vegetables and grains are produced from their own farmland, may have 4.36 fold more mercury intake per week than the allowed amount (1 μg/kg bw/week).
