# Supplementary material for: Mercury pollution in vegetables, grains and soils from areas surrounding coal-fired power plants
Source: Sci Rep. 2017 May 9;7:46545. doi: 10.1038/srep46545 (PMC5422849; doi:10.1038/srep46545)
Supplement: Supplementary Information [file srep46545-s1.pdf]

Supplementary Information

Mercury pollutions in vegetables, grains and soils from areas surrounding coal-fired power plants

Rui Li[1, #], Han Wu[1, #], Jing Ding[1], Weimin Fu[1], Lijun Gan[2] * & Yi Li[1, 3] *

#: these authors contributed equally to this work

1. College of Horticulture, Nanjing Agricultural University, Nanjing, 210095, P. R. China.
2. College of Life Sciences, Nanjing Agricultural University, Nanjing, 210095, P. R. China.
3. Department of Plant Science and Landscape Architecture, University of Connecticut, Storrs, CT 06269, USA

Correspondence and requests for materials

Correspondence and requests for materials should be addressed to Y. L. (yi.li@uconn.edu) and L. G. (ganlj@njau.edu.cn). Y.L. holds a 2 month/year visiting professor position at NAU.

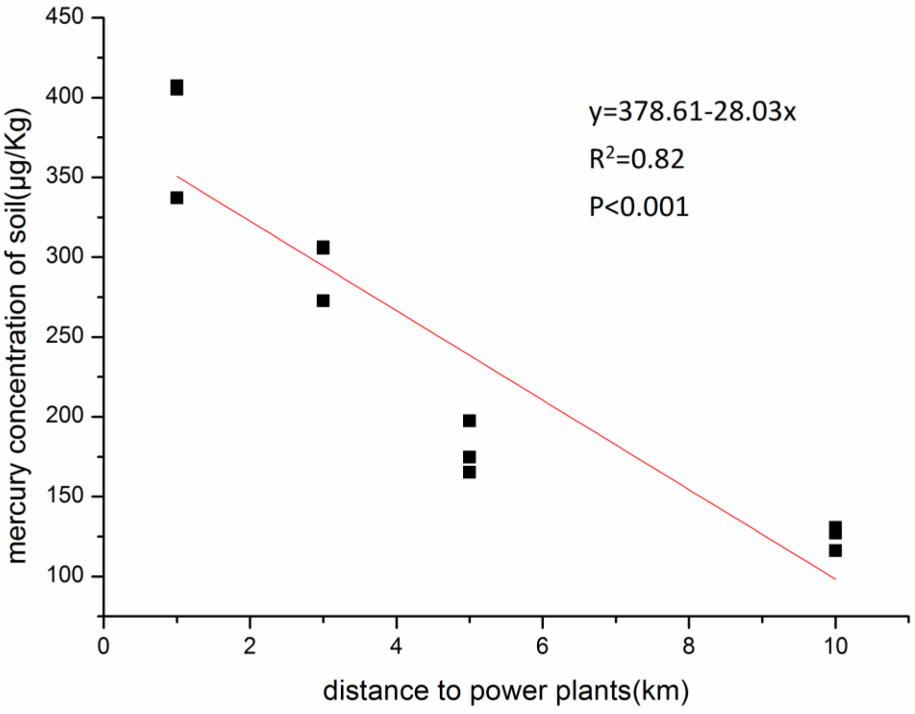

Supplemental Fig. 1: The mercury concentrations in the soil samples were negatively correlated with their distance to the studied coal-fired power plants

Samples	Mercury concentration (μg/kg)			
	Location B1	Location B2	Location B3	Location B4
Lettuce***	21.03±0.16	19.41±1.16	9.17±0.52	7.23±0.57
Amaranth**	29.29±5.06	7.50±0.21	5.52±0.86	3.64±0.37
Water spinach***	54.46±4.55	49.19±0.28	38.97±3.43	23.88±1.28
Tomato*	76.33±5.47	57.09±8.33	29.07±1.45	9.79±0.43
Eggplant*	43.36±1.71	25.02±1.80	14.61±2.95	3.25±0.41
Cucumber*	18.21±1.19	16.94±0.66	10.09±0.40	2.18±0.34
Cowpea**	57.30±9.24	21.75±1.55	18.46±0.61	11.20±0.95
Rice*	59.21±4.36	43.30±2.19	37.15±2.39	24.99±1.99
Soil	383.23 ± 32.59	294.91 ± 15.67	179.14 ± 13.53	124.58 ± 6.14

* = $P < 0.05$, ** = $P < 0.01$, *** = $P < 0.001$, the significance test was applied between samples with vegetable or grain samples and respective soil samples collected in the same field.

Supplemental table 1：The T-test result of mercury concentration vegetable and grain samples with soil samples
